# Role of the HPRG Component of Striated Muscle AMP Deaminase in the Stability and Cellular Behaviour of the Enzyme

**DOI:** 10.3390/biom8030079

**Published:** 2018-08-23

**Authors:** Francesca Ronca, Antonio Raggi

**Affiliations:** Laboratory of Biochemistry, Department of Pathology, University of Pisa, via Roma 55, 56126 Pisa, Italy; antonio.raggi@med.unipi.it

**Keywords:** AMP deaminase (AMPD), histidine-proline-rich glycoprotein (HPRG), striated muscle, Troponin T (TnT)

## Abstract

Multiple muscle-specific isoforms of the Zn^2+^ metalloenzyme AMP deaminase (AMPD) have been identified based on their biochemical and genetic differences. Our previous observations suggested that the metal binding protein histidine-proline-rich glycoprotein (HPRG) participates in the assembly and maintenance of skeletal muscle AMP deaminase (AMPD1) by acting as a zinc chaperone. The evidence of a role of millimolar-strength phosphate in stabilizing the AMPD-HPRG complex of both AMPD1 and cardiac AMP deaminase (AMPD3) is suggestive of a physiological mutual dependence between the two subunit components with regard to the stability of the two isoforms of striated muscle AMPD. The observed influence of the HPRG content on the catalytic behavior of the two enzymes further strengthens this hypothesis. Based on the preferential localization of HPRG at the sarcomeric I-band and on the presence of a Zn^2+^ binding motif in the N-terminal regions of fast TnT and of the AMPD1 catalytic subunit, we advance the hypothesis that the Zn binding properties of HPRG could promote the association of AMPD1 to the thin filament.

## 1. Introduction

AMP deaminase (AMPD) (EC 3.5.4.6) catalyzes the irreversible hydrolytic deamination of AMP to IMP and ammonia. Although the physiological function of AMPD is not yet fully understood, it appears to play a role in regulation of relative concentrations of intracellular purine nucleotide pools [1], the stabilization of adenylate energy charge [2], and the deamination of amino acids via the purine nucleotide cycle [3]. In skeletal muscle, the activity of the enzyme is much higher than in any other tissue [4], and it is well established that the ammonia produced by working muscle arises from AMP deamination [5]. Furthermore, the skeletal muscle enzyme (AMPD1) is activated during exercise, when the rate of ATP utilization exceeds the potential of the cell to resynthesize ATP. Because AMP deamination displaces the equilibrium of the myokinase reaction (2 ADP = 1 ATP + 1 AMP) toward ATP resynthesis, a proposed role for AMPD1 is to alleviate the exercise-induced decrease in the ATP/ADP ratio and its inhibitory effect on muscle contraction [6].

In a recent review [7], we have highlighted the relationship between AMPD1 and troponin T (TnT). During strenuous exercise, AMPD1 would undergo the proteolytic removal of its N-terminal domain causing the enzyme to show an unrestrained activity [7,8]. In vitro experiments carried out with rabbit AMPD1 showed that that behavior could be counteracted by the binding of the cleaved enzyme to the phosphorylated N-terminal region of fast rabbit TnT [9]. Since in native rabbit AMPD1 the 95-residue N-terminal domain holds the enzyme in a less active conformation due to the presence of a dinuclear zinc center connecting the N-terminal and C-terminal regions [10], it can be inferred that the interaction of the single catalytic Zn ion of the proteolyzed enzyme with the TnT N-terminal region that contains a putative metal binding site [7] restores the allosteric properties of AMPD1 that were removed by the limited proteolysis, i.e., inhibition by ATP. Together with the reported evidence that the reversible interaction between AMPD1 and myofibrils is promoted in vivo by intense muscle contraction [11,12], we suggest that the enzyme might mutually combine with TnT to fine tune the regulation of muscle contraction in fast muscle.

In this paper, we have summarized our previous observations that suggest that histidine-proline-rich glycoprotein (HPRG) is a subunit of AMPD1 that functions as a metallochaperone, and that a heterotetramer model for the AMPD/HPRG complex (i.e., a dimer of approximately 155 kDa heterodimers) can be envisaged [13]. On this basis, we now suggest that the presence of HPRG in the striated muscle AMP deaminase complexes (AMPD1 and AMPD3) should be taken into consideration in attempts to explain the previous controversial results obtained with AMPD preparations which were partially or totally deprived of the HPRG subunits. The evidence that the formation of a protein-protein complex between HPRG and the catalytic subunit of AMPD1 is critical for the stability of the enzyme, together with the preferential localization of HPRG at the regulatory thin filament in human skeletal muscle, allows one to advance the hypothesis of a HPRG-mediated interaction between AMPD1 and fast-twitch TnT.

## 2. Distribution and Localization of AMPD Isoforms

AMPD is found in all vertebrate tissues, and at least three main isoforms of the enzyme have been identified on the basis of immunological, physical, and kinetic properties [14]. Three major activities isolated from adult tissues and cells are termed isoform A (rat) or M (human) in skeletal muscle, isoform B (rat) or L (human) in liver, testes, and kidney, and isoform C (rat) in heart and erythrocytes, corresponding to the isoform E of human heart and erythrocytes [15,16]. The AMPD B/L and C/E enzymes are widely expressed, and tissues such as brain, lung, and spleen co-express more than one isozyme—probably combinations of the B-C/L-E isoforms [17,18,19]—whereas the isoform A/M expression is confined primarily to adult skeletal muscle.

Three AMPD genes (*AMPD1, AMPD2, AMPD3*) were cloned in rat and human, coding for AMPD A/M, AMPD B/L, and AMPD H/E, respectively, and different patterns of gene-specific mRNA abundance were observed in adult rat, rabbit, and human tissues [20,21]; this was in good agreement with published data regarding the expression of their encoded protein products. *AMPD2* mRNA is predominantly expressed at high levels in brain, and at low levels in other non-muscle tissues such as liver, thymus, placenta, pancreas, lung, spleen, kidney, aorta, uterus, and embryonic muscle [20,22]. Although a very low expression of AMPD2 was observed in heart and skeletal muscle, the two isoenzymes AMPD1 and AMPD3 are expressed simultaneously in striated muscle [23,24,25]. Prompted by a predicted homology between residues in the N-terminal domain of the AMPD3 polypeptides and the carboxyl-terminal ends of α-spectrin and fodrin, structural components of the cytoskeleton localized to the cytoplasmic surface of the erythrocyte membrane, AMPD E was found to be associated with the cytoplasmic surface of the erythrocyte membrane [26,27,28].

Studies in avian, rabbit, rodent, and human systems indicated the appearance of multiple forms of AMPD during the development of different tissues (reviewed in [14]). The embryonic isoform AMPD B is expressed in several organs of the perinatal and adult rat, i.e., spleen, lung, liver, thymus, and blood [29,30]. The fetal brain contains isozyme B as the predominant form, and after birth, the synthesis of isozyme C subunit continues to increase to show five isoenzymes in adult rat [30]. Both liver and heart have five isozymes in early postnatal life. During postnatal development, a continuous shift to the B isozyme occurred in liver and kidney, whereas a shift to the isozyme C was observed in heart [17]. Similar changes in the AMPD activities were also measured in several tissues i.e., heart, brain, erythrocytes, and skeletal muscle during chick ontogenesis [31,32,33]. Rat striated muscles express more than one AMPD isoform during muscle development. The AMPD B isoform is expressed in the embryonic muscle, whilst the heart isoenzyme (AMPD C) is encoded in the perinatal muscles. At or just before birth, a third isoform of AMPD appears, corresponding to the main isoform of the adult skeletal muscle [14]. The isozyme transition was also found during development of hen heart, since the adult heart isoform is different than the embryo one [34].

Switching in the expression with time courses similar to those for AMPD [14] has been observed also for other proteins during muscle development, i.e., myosin heavy and light chains, actin, tropomyosin, troponin, and creatine kinase isoforms (reviewed in [14]), suggesting that common developmental signals may coordinate expression of these proteins during myocyte development.

*Ampd1* mRNA is relatively more abundant in glycolytic fast-twitch (type II) fibers than in the oxidative slow-twitch (type I) fibers of skeletal muscles of the rat and rabbit, where *Ampd3* mRNA is more abundant than *Ampd1* mRNA [29,35]. In the mixed-fiber adult human skeletal muscle and in the heart, both the *AMPD1* and *AMPD3* genes are expressed, the *AMPD3* mRNA being relatively more abundant than the *AMPD1* one [35,36].

A 4–10-fold higher level of AMPD activity in white muscle compared to that in red muscle was documented in different species [37]. Qualitative dissimilarities have also been demonstrated between the preparations of AMPD from the different types of striated muscle. By chromatography on cellulose phosphate of extracts of rabbit red muscles, two peaks of AMPD activity were obtained (forms A and B), the position of the former corresponding to that of the single form present in the heart, and the latter having the same chromatographic properties as the single form present in white muscles [38]. Form B is inhibited by ATP, as is observed with white muscle AMPD, whereas form A is not affected by the nucleotide [39]. Different chromatographic patterns of AMPD isoforms were also obtained with human muscle extracts. With human pectoralis major, two peaks of AMPD activity were obtained, both in the elution region of isoenzyme B [38], whereas the preparation from quadriceps femoris gave a major peak of activity in the elution region of isoform A that was, however, inhibited by ATP, indicating the presence of the white muscle isoform [40].

AMPD immunohistochemistry in human skeletal muscle confirmed the distribution of the AMPD isoenzymes based on the fiber type composition, and indicated that the three main isoforms are localized in different parts of muscle tissues [41]. AMPD M was localized predominantly in Type II (fast-twitch) fibers, where it displayed a cross-striation pattern, and was primarily concentrated subsarcolemmally and intermyofibrillarly. Interestingly, AMPD M was also strongly concentrated at the neuromuscular junctions co-localizing with acetylcholine receptor clusters and in capillary endothelial cells. AMPD E was localized mainly in Type I (slow-twitch) fibers showing a cross-striation pattern, and to a lesser extent, in smooth muscle cells, capillary endothelium, red blood cells, and in nerve bundles. AMPD B was concentrated within non-myofiber elements of muscle tissue.

It was previously reported that a histidine-proline-rich-glycoprotein (HPRG)-like protein associates with rabbit skeletal muscle AMPD that can be assumed to represent the white-muscle AMPD1 isoform [42]. The solubility of the catalytic subunit of the enzyme was shown to be markedly reduced when the HPRG component was separated by zinc-affinity chromatography [43]. The hypothesis that HPRG functions as a metallochaperone in the formation of a heterodimer with AMPD1 is strengthened by the experimental evidence that rabbit HPRG hosts two Zn ions coordinated by His residues and sulfur from cysteine residues [44]. Comparison of the primary structure of a proline-rich region of HPRG of several mammalian species shows the presence of a conserved binding site that might coordinate the Zn ion with an amino acid arrangement compatible with the cysteine-containing site that has been identified experimentally for rabbit HPRG [45]. It was also shown by Sabbatini et al. [46] that rabbit skeletal muscle cells do not transcribe HPRG, but internalize the protein from serum, in agreement with the demonstration that HPRG mRNA is produced only in the liver [47]. Recent in vivo experiments [48] have shown that after intravenous injection, HPRG internalized quickly in healthy tissues, including muscle, and in tumors. Interestingly, it was earlier reported that chicken muscle AMPD is rapidly cleared from the circulation of chickens after intravenous injection of the purified enzyme [49], primarily by parenchymal cells of the liver through the interaction with cell-surface heparan sulphate [50]. The existence of a HPRG component in the AMPD structure, together with the well-known interaction of HPRG with heparin [51], give a molecular basis of the above observations. To date the physiological role of HPRG in plasma has not clearly been determined, as it appears to be involved in many processes, such as blood coagulation and fibrinolysis, immune complex clearance, cell adhesion, cell migration, and the transport of metal ions, due to its ability to bind various ligands such as phospholipids, fibrinogen, plasminogen, heparin, heparan sulfate, tropomyosin, heme, and divalent metal ions (reviewed in [47]).

Comparison of serial sections of healthy human skeletal muscles, after the AMPD histoenzymatic stain and the HPRG immunostaining, showed that the immunopositivity of HPRG in Type IIB fibers was always associated with high levels of AMPD activity, whereas Type IIA and Type I fibers always gave a weak response to the HPRG antibody, even in the specimens where Type IIA fibers showed an AMPD histoenzymatic staining similar to that of IIB, suggesting a preferential association of HPRG to the AMPD isoenzyme contained in fast-twitch glycolytic fibers [40]. A recent immunohistochemical study with human gastrocnemius and quadriceps femoris has shown a localization of HPRG at the I-band level, where it shows the same distribution of actin and where AMPD1 is present in major concentration [52].

## 3. Human Skeletal Muscle AMPD Deficiency. Correlation between the Level of Enzyme Activity and the Muscle Content of HPRG

It has been reported that different mutations may cause deficiency of human AMPD M; among them, the most recurrent (2% of randomly selected muscle biopsies) is the nonsense mutation C34T in the exon 2 of the AMPD1 gene [53]. From a clinical point of view, this deficiency is characterized by muscular weakness, exercise-induced myalgia and cramping after exercise, and by low residual AMPD activity (usually less than 2% of control mean). Since most subjects are asymptomatic, some investigators concluded that this disease is a harmless polymorphism [54,55,56,57]. A Secondary deficiency of AMPD M has also been associated with many pathological conditions, including myopathies, neuropathies, and motor neuron diseases [58]. Subsequentially, an investigation on the genetic characteristics of AMPD M deficiency in the Dutch population demonstrated the same underlying molecular defect, a C34T transition, in both types of deficiency, suggesting that the Secondary form is a Coincidental AMPD deficiency [54].

It has been proposed that alternative splicing of exon 2 of the AMPD1 gene (that removes the mutation from 0.6 to 2% of the AMPD transcripts) in individuals who have inherited the C34T mutation could provide a mechanism for phenotypic rescue, because these subjects could produce a small amount of fully functional AMPD M that, together with the AMPD3 expressed in oxidative fibers, could lead to the production of an active AMPD1/AMPD3 enzyme [59,60]. Moreover, a change of subunit composition of AMPD was previously observed in rat skeletal muscle in response to changes in *Ampd1* and *Ampd*3 gene expression induced by denervation [61]. In denervated rat muscle, an increase in *Ampd1* mRNA that excludes exon 2-encoded sequences was accompanied by an increase in *Ampd3* expression. Although the data of the literature show that in patients affected by the Primary AMPD deficiency the isoform E is expressed at a normal level, as evidenced by Northern blot analysis and immunohistochemistry [21,36,41], significant amounts of residual AMPD activity were removed by anti-E serum from skeletal muscle biopsies prepared from individuals with an inherited AMPD M deficiency, suggesting that an increased expression of AMPD3 isoforms might compensate the reduced or null AMPD M activity [41].

We have reported that the immunological reaction of the anti-HPRG antibody and of anti-AMPD (isoform M) antibody in human skeletal muscle biopsies from patients with AMPD deficiency clearly indicated a correlation between the muscle content of HPRG protein and the level of AMPD activity [62]. The patients affected by Primary and Coincidental AMPD deficiency, which were characterized by an absence of enzyme activity and AMPD immunoreactivity, showed no HPRG immunoreactivity, whereas in all of the biopsies of the patients, heterozygous for the C34T mutation (acquired AMPD deficiency), which exhibited an AMPD activity detectable in solution, were positive to the anti-HPRG antibody. The interpretation of the significance of these observations suggests a physiological mutual dependence between skeletal muscle HPRG and AMPD polypeptides with regard to their stability.

## 4. A Highly Differentiated N-Terminal Region of AMPD Is Produced by Alternative Splicing Events

AMPD coding sequences have been highly conserved during evolution, and it is likely that the three *AMPD* genes arose from duplication of a common primordial gene [63], and subsequently, acquired differences via divergent evolution. Whilst the C-terminal region of the enzyme is conserved from yeast to vertebrates and contains the catalytic Zn, the N-terminal region differentiates among the isoforms.

Primary amino acid sequence alignments identify divergent N-terminal and conserved C-terminal domains across human AMPD isoforms [25,36,63]. In addition, all three human *AMPD* genes produce multiple transcripts that encode additional variation at or near the N terminus of each isoform [21,64,65]. The three AMPD polypeptides share a similar 550 amino acid C-terminal end (62–70% identical [21,36]) that contains a motif SLSTDDP believed to take part in the constitution of the Zn binding catalytic center of the enzyme. The association of evolutionarily conserved amino acid residues with enzymatic deamination extends from bacterial adenosine deaminase to mammalian adenosine deaminases and eukaryotic AMP deaminases [66]. Conversely, each AMPD polypeptide differs by divergent N-terminal sequences of 200–330 amino acids with less than 36% identity to each other. In addition, differential promoter use and alternative splicing add extensions or substitutions of four amino acids in AMPD1 [23,64], 47–128 amino acids in AMPD2 [65], and 7–9 amino acids in AMPD3 [36] at the distal N-terminal end of each AMPD polypeptide. Each of the three human genes produces more than one transcript through the use of multiple promoters and/or alternative splicing events. The *AMPD1* gene produces two mRNAs as the result of a cassette-type alternative splicing event involving the miniexon 2 [64]. The *AMPD2* gene generates three mRNAs by employing two promoters (1A and 1B) and a cassette-type alternative splicing event involving exon 2 in type 1B transcripts only [67]. The *AMPD3* gene produces at least four mRNAs by using three separate promoters (1a, 1b, and 1c) that differ for the length of the transcripts, the 5′-nucleotide sequence, and the N-terminal amino acid sequence [36].

It has been reported that divergent N-terminal residues may play an important role in the intracellular distribution of AMPD as evidenced by their effects on contractile protein binding behavior [68]. A stretch of sequence in the unique N-terminal region of the AMPD1 polypeptide is required for the high actomyosin binding capacity of isoform M [69]. This observation is functionally significant because contractile protein binding is an important physiological regulator of the catalytic activity of AMPD1 in stimulated skeletal muscle [70,71]. It has also been shown that the first 48 amino acids of the N-terminus of isoform E, that is highly expressed in skeletal muscle type I fibers [41], dramatically suppress contractile protein binding capacity of this enzyme [69], a behavior that could facilitate other intracellular interactions. More recently, a series of studies has shown that interactions between purified pig heart AMPD, the porcine ortholog of human isoform E [72], and isolated cytoplasmic membrane vesicles and artificial lipid bilayers [73,74,75], alters secondary structure and regulatory behavior of the enzyme. The association of the N-terminal domain of AMPD3 with the cytoplasmic face of erythrocyte ghost membranes is accompanied by reduced catalytic activity of the enzyme [27]. Taken together, these observations suggest that divergent N-terminal domains in each AMPD polypeptide contribute to the isoform-specific behaviors of the enzyme [76] that might be based on the presence of an additional Zn ion with regulatory function [77].

The nature of AMPD1 as a zinc-containing metalloenzyme is well documented [78,79]. The presence of 2.0 and 2.6 g atoms of zinc was established, respectively, per mole of rat enzyme (molecular weight (MW) 290 kDa) and rabbit enzyme (MW 278 kDa). The rabbit apoenzyme binds 4 g atoms of zinc per mol, but the increase of Vmax due to the addition of the fourth zinc atom is only 28% of that expected. This suggests that the fourth zinc atom is not directly associated with activity [79]. Moreover, alignment of the amino acid residues supposed to be in contact with the single zinc ion of the yeast AMPD subunit [80] with the predicted amino acid sequence for vertebrate AMPD1 demonstrates conservation of only three amino acid residues located in the C-terminal region of the enzyme, suggesting that the model of a penta-coordinated zinc bound at the catalytic site of yeast AMPD cannot be unambiguously extended to AMPD1 [13].

Evidence was recently provided for a dinuclear zinc site in rabbit skeletal muscle AMPD1 compatible with a (μ-aqua) (μ-carboxylato) dizinc(II) core with an average of two histidine residues at each metal site. The two Zn ions in the AMPD1 metallocenter would operate together as a single catalytic unit, but with independent function, one of them (Zn_1_) acting to polarize the nucleophile water molecule, whilst the other (Zn_2_) acts transiently as a receptor for an activating substrate molecule. The putative Zn_2_-binding site, localized between the 51–60-residue region (HHEMQAHILH), might represent the regulatory site responsible for the positive homotropic cooperativity behaviour of AMPD1 [77]. N-terminal analysis of the peptides liberated by limited tryptic digestion of different enzyme preparations suggested the existence of two different protein conformations consistent with the presence of the Zn_2_ ion connecting the N-terminal and C-terminal regions of AMPD1 [10,13].

Since the putative Zn_2_ binding site contained in the N-terminal region of the enzyme is removed by the proteolytic process that occurs during storage of the enzyme, earlier data on the metal analysis of rabbit AMPD1 is likely to be very imprecise because the enzyme preparation used for those studies did not contain this N-terminal region.

To date, no crystal structures are presently available for the native form of AMPD1, since the published X-ray crystal structures of AMPD are only those of N-terminally truncated rabbit AMPD1 [81] and of its plant ortholog encoded by Arabidopsis embryonic factor 1 (FAC1) [82]. The crystal structure of the complex of the two enzymes with specific inhibitors, based around coformycin, showed a coordination of the catalytic zinc ion by three histidine and one aspartic acid residues, which is consistent with the metallocenter that was described for mouse adenosine deaminase (ADA) [83], and was also proposed for the monomer of yeast AMPD [80]. It was therefore suggested that AMPD1 shares with ADA a conserved catalytic mechanism. In our opinion, however, the presumed homology between rabbit AMPD1 and mouse ADA metallocenters might stand only for the proteolysed AMPD which, as a consequence of the removal of residues 1–95, is deprived of the Zn_2_ ion connecting the N-terminal and C-terminal regions of native AMPD1. It is likely, therefore, that the N-truncated protein could only bind the Zn_1_ ion with a coordination that differs from what has been described for Zn_1_ in the dinuclear zinc center [10]. Further studies are necessary to establish which domains of HPRG are required for contact with AMPD, and whether a mechanism exists for transferring zinc between the two proteins. However, it has been reported that no zinc is bound to HPRG when the ternary complex of HPRG with Zn and the AMPD catalytic subunit is formed [10]. This conclusion is supported by our previous observation [84] that the removal of zinc by EDTA from rat AMPD1 does not abolish the tetrameric complex, since the apoenzyme behaves as a species with an apparent molecular mass of 254 kDa, which is somewhat lower than that of 285 kDa calculated for the native enzyme, suggesting that the variation observed might be due to an enzyme conformation change (see Chapter 6, Figure 3C).

## 5. The Inherent N-Terminal Proteolysis of Striated Muscle AMPD Isoforms

The reported molecular mass of the two striated muscle AMPD isoform subunits, purified from different animal sources by the method of Smiley et al. [85], are consistently lower than those predicted from the cDNA sequences (Table 1).

For example, highly conserved rabbit, rat, and chicken *Ampd1* cDNAs predict polypeptides with molecular masses of 85–86 kDa [86], whereas AMPD1 purified from rabbit [44,87,88], rat [17,84,89,90,91], and chicken [87,88] skeletal muscle exhibit subunit molecular masses ranging from 60 to 85 kDa, the smaller size being determined when the enzyme was purified from frozen tissues. Based on the cDNAs sequences, the AMPD3 polypeptides would have subunit molecular masses of 92–94 kDa, whilst the subunits of the purified enzymes show molecular weights that are about 10 kDa lower than the predicted ones [92,93,94,95]. The reason for these discrepancies between the observed and the predicted molecular masses of striated muscle AMPD isoforms are attributable to proteolysis occurring during purification and storage of the enzymes. As a matter of fact, larger subunit molecular masses were obtained when purifications were performed in the presence of protease inhibitors, but subsequent degradation occurred upon storage [96,97].

Although previous attempts to avoid degradation of purified skeletal muscle AMPD by using various inhibitors of proteolysis have met with little or no success, it has been shown that leupeptin and E-64, both inhibitors of calpains, were fully effective at limiting in vitro proteolysis of three variants of human liver recombinant AMPD [98], suggesting that calpain might be a potential processor in vivo of the enzyme. Recombinant expression of human AMPD1 and AMPD3 isoforms produced enzymes with apparent full-sized subunits [60]. However, storage of these purified preparations at 4 °C yielded subunits lacking N-terminal residues, since AMPD1 and AMPD3 recombinant isoenzymes were proteolysed to truncated polypeptides ΔI86 and ΔH98, and ΔL88 and ΔM90, respectively, all cleavages sites having Leu at P2, strengthening the hypothesis that a calpain-mediated proteolytic cleavage [99] plays a significant role in the regulation of AMPD activity in striated muscle.

We were able to purify from fresh rabbit white muscle tissue an AMPD1 showing an 85 kDa subunit which is consistent with the mass predicted from the cDNA sequence (Table 1), whilst we obtained a proteolysed 75 kDa subunit core from frozen muscle or as effect of storage of the freshly prepared enzyme at 4 °C [96]. We have reported that a proteolytic activity is associated with purified rabbit white muscle AMPD1, showing a substrate specificity which is similar to that of the ubiquitous calpains, μ- and m-calpain, requiring a hydrophobic amino acid residue in the P2 position, with a strong preference for Leu over Val [100]. The rabbit N-terminal proteolysed enzyme is more active at low substrate concentration, and no longer inhibited by ATP, similarly to the effect of the removal of the 95 aa N-terminal domain by trypsin [101]. Although the function and the mechanisms of calpain proteolysis of AMPD are unknown, it has been hypothesized that this phenomenon could be responsible for the large level of ammonia accumulation during episodes of strong tetanic contraction of skeletal muscle or during rigor mortis [8]. Interestingly, the rabbit AMPD1 N-terminal domain contains a calpastatin-like sequence that might protect against an unrestrained activation of the enzyme that otherwise would lead to the depletion of adenine nucleotide store [100].

It has been shown that a histidine-proline-rich glycoprotein is strongly associated with freshly purified rabbit AMPD1 [13,42]. Since we observed that the rate of fragmentation of HPRG-enriched AMPD1 on storage was reduced, we suggested that the cystatin domain at the N-terminus of HPRG component would protect AMPD1 against fragmentation by thiol proteases [44].

A tetrameric structure has been suggested for AMPD1. However, a wide range of molecular masses (from 238 to 326 kDa) has been reported for the enzyme isolated from various species (reviewed in [13]), that can be interpreted as being due to the inherent instability of the enzyme during the prolonged extraction steps. Our determination by sedimentation-equilibrium analysis of the molecular mass of freshly prepared rabbit AMPD1 indicated the presence of two species of 173 kDa and 309 kDa, which were interpreted as being consistent with the existence of a dimer-tetramer equilibrium of two 85 kDa catalytic subunits assembled with two approx. 70 kDa HPRG subunits [13]. The 222 kDa molecular mass we determined for the enzyme after its limited proteolysis by trypsin can be explained by taking into account our observation that the process also liberates from the HPRG component a 30 kDa fragment [44], thereby suggesting a tetramer assembly for proteolysed AMPD1 formed by two approx. 75 kDa N-truncated catalytic subunits and two approx. 40 kDa proteolysed HPRG subunits. In agreement with the new model for AMPD1 as a 1:1 molecular adduct composed by one catalytic dimer and one HPRG dimer, we have shown that an anti-HPRG antibody selectively marked the type IIB fibers that contain the highest level of AMPD isoform M [40]. Furthermore, the immunological reaction of the anti-HPRG antibody and of an antibody specific to AMPD (isoform M) in human skeletal muscle biopsies from patients with AMPD deficiency clearly indicated a correlation between the muscle content of the HPRG protein and the level of AMPD activity [62].

## 6. Importance of Inorganic Phosphate for the Stability of the AMPD-HPRG Complex

Previous controversial results were obtained with AMPD preparations partially or totally deprived of the HPRG subunits.

It was reported by Smiley et al. [85] that the purification of AMPD1 from frozen rabbit skeletal muscle by cellulose-phosphate ion exchange chromatography yielded the enzyme in higher yields and of higher specific activity when the data were compared with those reported for the purification of the enzyme via its dissociation by inorganic phosphate from actomyosin preparations [102]. It should be noted that comparative studies made by Currie and Webster [102] on various preparations from both rat and rabbit fresh muscle showed that the phenomenon of the dissociation of rat AMPD1 by precipitation of actomyosin solutions at low ionic strength in the presence of inorganic phosphate did not occur with myosin or actomyosin from the rabbit. In this context the observation that rat AMPD1 has an increased susceptibility to proteolysis when compared to the rabbit enzyme deserves consideration. Rat AMPD1 subunit is routinely isolated as a proteolysed fragment with values of apparent molecular weight ranging from 60 to 70 kDa, whilst AMPD1 from fresh rabbit muscle yields a native 85 kDa peptide, even in the absence of protease inhibitors (Table 1). In light of this observation, it can reasonably be assumed that the higher affinity for the myofibril proteins shown by rabbit AMPD1 in comparison with the rat enzyme might be due to the higher stability of the N-terminal region of the enzyme presumably involved in protein-protein interactions with the myofibrils.

**Figure 1:**
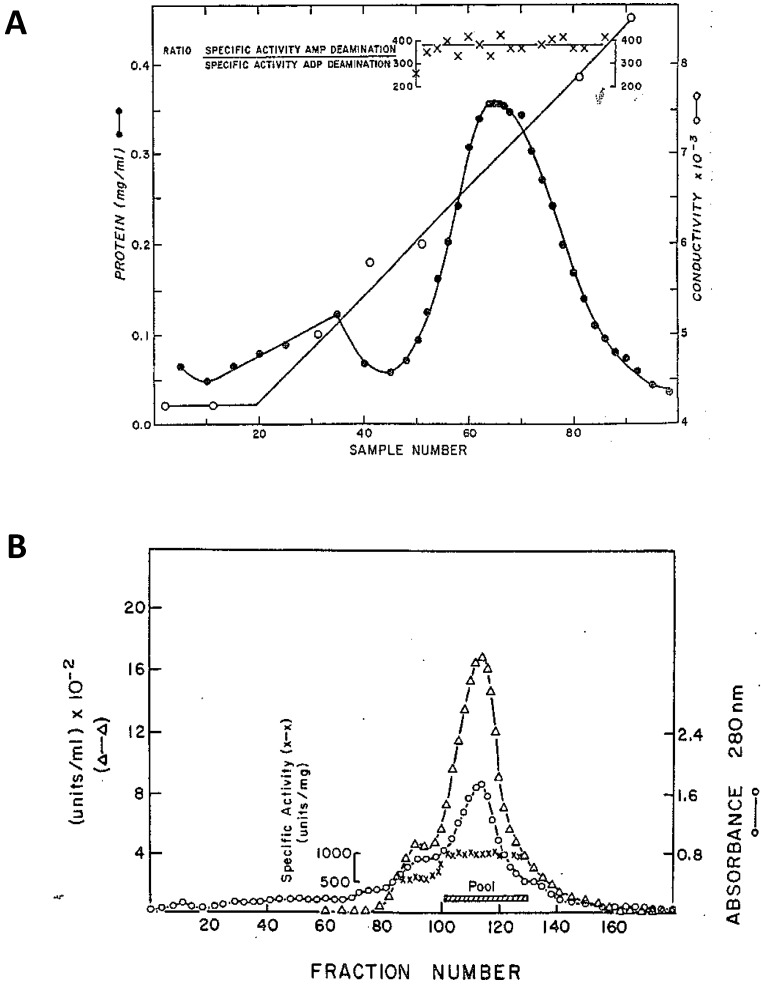
Elution profiles of AMPD1 on cellulose phosphate. (**A**). Rabbit AMPD1 according to Smiley et al. [85]. The enzyme was eluted with a linear gradient of 0.54–1.0 M KCl containing 1 mM β-mercaptoethanol at pH 7.0. Fraction protein content (●) and enzyme activity (x) are reported. (**B**). Rat AMPD1 according to Coffee and Kofke [90]. The enzyme was eluted with a linear gradient of 0.45–1.0 M KCl containing 2 mM β-mercaptoethanol at pH 8.0. Fraction protein content (○), enzyme activity (Δ) and specific activity (x) are reported.

Smiley et al. [85] reported that AMPD1 from frozen rabbit muscle remained bound to cellulose phosphate under conditions (0.45 M KCl, pH 7.0) at which apparently not other proteins are bound and the subsequent elution with 1.0 M KCl, pH 7.0, yielded an apparently homogeneous preparation of the enzyme. However, the chromatographic profile of AMPD1 eluted from the cellulose-phosphate resin by linear increasing concentrations of KCl showed the presence of a small protein peak preceding the main activity peak eluted at about 0.8 M KCl (Figure 1A). That minor peak was not examined. The results obtained by Coffee and Kofke [90] when the cellulose-phosphate chromatography procedure was applied to rat AMPD1 (Figure 1B) are similar to those above described for rabbit AMPD1. The main peak of enzymatic activity was preceded by a minor component with lower specific activity that was discarded. The same authors, by a subsequent fractionation on Diethylaminoethyl -cellulose and a second step of purification on gel filtration, yielded a homogeneous enzyme with a subunit molecular weight of 60 kDa that, however, quickly lost the enzymatic activity.

**Figure 2:**
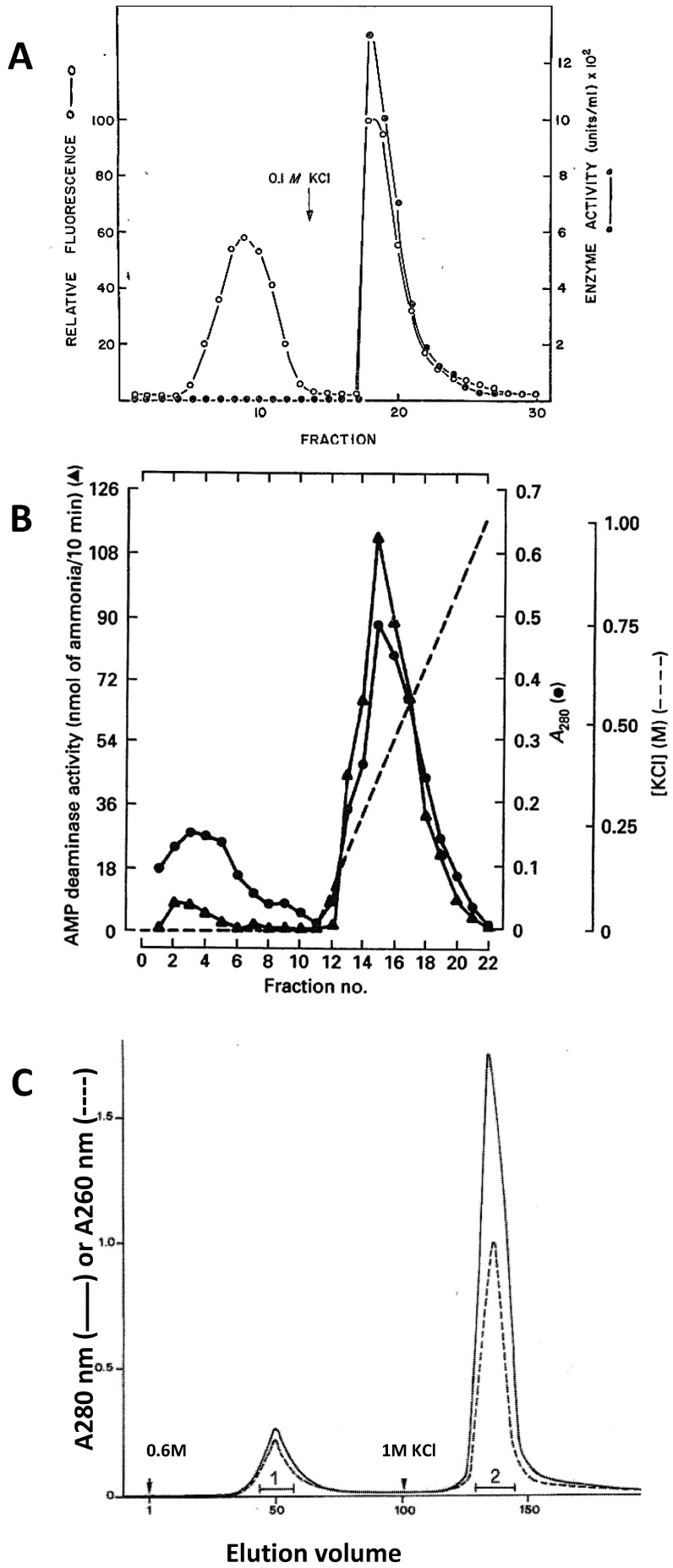
Chromatography attempts to improve the purification of AMPD1 and AMPD3. (**A**). Affinity chromatography of rat AMPD1 [91]. After separation on cellulose-phosphate, AMPD1 was applied to an AMP-Sepharose column and washed with 20 mM potassium phosphate (pH 7.5) followed by 20 mM potassium phosphate (pH 7.5) containing 0.1 M KCl. The fractions protein content was assessed by fluorescence (○). (**B**). Affinity chromatography of rabbit heart AMPD3 [92]. After cellulose-phosphate column and Sephacryl-300 column fractionation, AMPD3 was applied to an AMP-Sepharose column and eluted with a linear gradient of 0.0–1.0 M KCl (- -) in 90 mM potassium phosphate (pH 6.5) containing 0.1% *v*/*v* β-mercaptoethanol. (**C**). Fractionation of rabbit AMPD1 by stepwise elution on cellulose-phosphate [10]. The enzyme was eluted by two successive steps with 0.6 M KCl, pH 7.0, and 1 M KCl, pH 7.0, as indicated by the arrows. Protein content was assessed by measuring A_280_ (solid line) and A_260_ (dashed line). The enzyme activity determined on the pooled fractions 1 and 2 measured 0.5 units/mg and 210 units/mg for pool 1 and 2, respectively.

Further attempts to improve rat AMPD1 purification by adding an affinity chromatography step on an AMP-Sepharose column did not eliminate an additional proteic component void of catalytic activity (Figure 2A). The results obtained during the purification of rat heart AMPD3 described by Thakkar et al. [92] (Figure 2B) are similar to those reported above for rat AMPD1. After elution from cellulose-phosphate with 1 M KCl, the concentrated extract was applied to a Sephacryl S-300 column and eluted with a phosphate buffer (pH 6.5) containing 0.18 M KCl and 0.1% *v*/*v* β-mercaptoethanol. The pool of the active fractions was submitted to affinity chromatography on an AMP-Sepharose column. The major AMPD3 polypeptide with 81 kDa apparent molecular weight was eluted with 0.5 M KCl. A minor proteic component with an apparent 71 kDa molecular weight and negligible catalytic activity was eluted at 0.0 M KCl, and was not analysed further.

The constant presence of presumably the same additional peptide found by all the authors mentioned above was also observed in our laboratory in the AMPD1 preparation from fresh rabbit muscle. In order to better isolate the contaminant peptide, we introduced a modification in the cellulose phosphate chromatography which consisted of a stepwise elution of the enzyme that remained bound to cellulose phosphate after having washed the column with 0.45 M KCl. The two successive washing steps with 0.6 and 1.0 M KCl effectively separated the minor peptide from the purified enzyme (Figure 2C) [44]. Further investigations of the fractions eluted with 0.6 M KCl revealed that the peptide previously discarded as a contaminant of the enzyme preparation could be identified as the HPRG component of rabbit AMPD1 [13,44]. However, since the same HPRG N-terminal sequence (LTPTDXK) was found in both elution peaks whilst the AMPD specific activity in peak 1 was negligible in comparison with that of the protein in peak 2, it can be assumed that the 1 M KCl-eluted enzyme can be taken as the whole AMPD1 (i.e., the HPRG/AMPD protein-protein complex), and that 0.6 M KCl selectively elutes the HPRG component from the AMPD1 complex adsorbed to cellulose phosphate, giving rise to a complex with an extremely high HPRG/AMPD molar ratio. Analysis by SDS/PAGE of the two peaks showed that both gave rise to a main band of approximately 85 kDa, corresponding to both the catalytic subunit and the HPRG component and an additional faint 95 kDa band [44]. With aging, the 85 kDa catalytic subunit was transformed to an approximately 70 kDa core, resistant to further proteolysis [96,103]. In contrast, the 85-kDa band given by HPRG eluted with 0.6 M KCl was almost completely transformed in a 95 kDa band that was resistant to proteolysis [44]. It should be noted that rabbit plasma HPRG (58 kDa) migrates in SDS/PAGE with an apparent molecular mass of 95 kDa higher than that calculated, taking account of its carbohydrate content (70 kDa), as a result of the reduction of the disulfide bond connecting Cys-6 and Cys-509 [45]. Therefore, the heterogeneity observed in sedimentation-equilibrium centrifugation of freshly prepared rabbit AMPD1 in 1.0 M KCl, pH 7.0, that yielded two species of 173 and 309 kDa [104] could be interpreted as being due to the presence of HPRG/AMP deaminase protein-protein complexes with a different molar ratio, the observed 309 kDa molecular mass determined for the heavier component being in agreement with a model for AMP deaminase quaternary structure in which two 85 kDa catalytic subunits assemble with two approximately 70 kDa HPRG subunits.

Thakkar et al. [105] showed that SDS/PAGE of rabbit heart AMPD3 purified under phosphate-free conditions gave rise to three bands of approx. 80 kDa, 70 kDa, and 50 kDa. A similar pattern was obtained when AMPD3 purified in the presence of phosphate that, in untreated form, migrated as a single band of approx. 80 kDa was incubated with 2 µg of trypsin for 5 min. More intensive proteolytic treatment (20 µg trypsin for 30 min) enhanced the number and intensity of the bands (80, 60, 55 and 50 kDa) obtained with rabbit heart AMPD3 purified in the presence of phosphate. A quite different SDS/PAGE picture was observed as an effect of trypsinization of rabbit AMPD3 prepared without phosphate: the presence of single bands of 80 kDa and 60 kDa being observed when the enzyme was incubated with 2 µg of trypsin for 5 min and 20 µg trypsin for 30 min, respectively.

Moreover, rabbit heart AMPD3 isolated with phosphate assumed the kinetic and regulatory properties of the phosphate-free enzyme upon dialysis against phosphate-free buffer, whereas the reverse did not occur. As effect of the absence of phosphate, rabbit heart AMPD3 was no longer activated by ATP and ADP, and showed a hyperbolic curve of the enzyme activity as function of substrate concentration, whereas that obtained with the enzyme purified in the presence of phosphate was sigmoidal. On the basis of these observations, the authors suggest that the cardiac AMPD isoforms previously described may represent pseudo-isoenzyme species generated by an irreversible molecular alteration of one basic tissue-specific isoform caused by the absence of phosphate [105]. However, all these data could be now interpreted considering that the preparation of rabbit heart AMPD3 without phosphate yields the catalytic subunits of the enzyme dissociated from the HPRG subunits that are lost during the purification.

**Figure 3:**
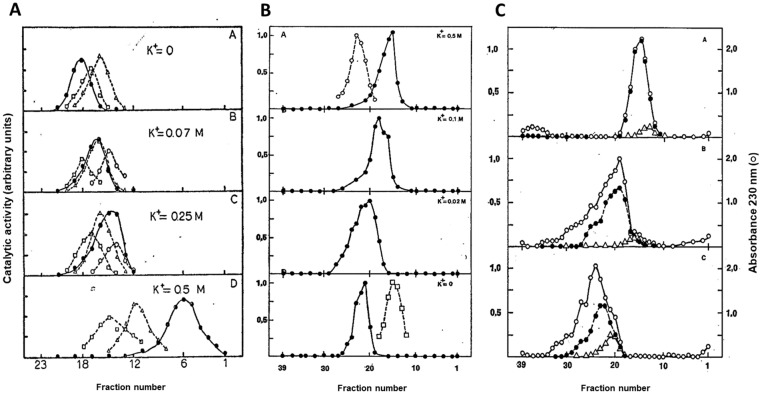
Comparison of sedimentation of rabbit and rat AMPD1 in sucrose density gradients. (**A**). Activity profiles of rabbit AMPD1 (●) and marker enzymes: lipoamide dehydrogenase (□), pyruvate kinase (○) and alcohol dehydrogenase (Δ), in sucrose density gradients in the absence of KCl (A) or in the presence of the indicated concentrations of KCl (B-D) [106]. (**B**). Activity profiles of rat AMPD1 (●) and marker enzymes: lactate dehydrogenase (○) and catalase (□), in sucrose density gradients in the absence of KCl (D) or in the presence of the indicated concentrations of KCl (A–C) [84]. (**C**). Sedimentation of EDTA-treated rat AMPD1 in sucrose density gradients containing 0.1 M KCl/50 mM potassium phosphate buffer (pH 6.5) (A) or a buffer containing 0.5 M KCl (B) or 0.02 M KCl (C) in the absence of phosphate. The enzyme was treated with EDTA until inactivations of 90% (A and B) and 70% (C) were observed. The fractions protein content was assessed by measuring A_230_ (○). The catalytic activity was determined in the presence of 1 mM EDTA (Δ) or 15 μM ZnCl_2_ (●) [84].

The presence of the HPRG subunit as a component of AMPD1 allows us now to reinterpret the data of the literature obtained with the sedimentation of the enzyme in sucrose density gradients at different concentration of KCl. It should be noted that the kinetic and regulatory properties of AMPD1 are strictly dependent on KCl concentration, and that K^+^ is the most effective activator of AMPD1 among monovalent cations [85,106]. In fact, at low concentrations of KCl, the enzyme follows sigmoid kinetics, with a Hill coefficient of about 1.4–1.9, suggesting the homotropic cooperativity between two catalytic subunits. The addition of 50–100 mM KCl reverts the curve to a hyperbolic one [106]. It was hypothesized that rabbit AMPD1 exists in a reversible and K^+^ dependent equilibrium between a structure composed of four AMPD1 subunits and one composed of two AMPD1 subunits, since the enzyme showed approx. molecular weights of 280 kDa at 0.5 M KCl (Figure 3A, box D) and of 150 kDa at 0.07 M KCl (Figure 3A, box B) [106].

However, an asymmetrical peak was obtained at the intermediate KCl concentration of 0.25 M (Figure 3A, box C), suggesting that the equilibrium between tetramer and dimer might be due to the association of subunits with heterogeneous molecular weight. In the absence of KCl, and at protein concentrations less than 1 mg/mL, rabbit AMPD1 sedimented as a monomer of 81 kDa molecular weight (Figure 3A, box A). The study of the aggregation state of rat AMPD1 with increasing concentrations of KCl (Figure 3B) gave results similar to those obtained with rabbit AMPD1 [84]. The enzyme sedimented as a symmetrical peak at the position of 239 kDa molecular weight in the presence of 0.5 M KCl (Figure 3B, box A), whereas at a lower concentration of K^+^, the peaks were clearly asymmetrical and corresponded to lower molecular weights, ranging from 200 kDa at 0.1 M KCl (Figure 3B, box B), 190 kDa at 0.02 M KCl (Figure 3B, box C) and 176 kDa in the absence of KCl (Figure 3B, box D), without the evidence of the existence of the AMPD1 monomer found by Ashman and Atwell [106] in rabbit AMPD1 (Figure 3A, box A). At the end of the sedimentation at all K^+^ concentrations, rat AMPD1 retained full activity, and the protein gradient profile coincided with the activity profile.

The results of the sedimentation experiments carried out with rat AMPD1 were interpreted as being due to the existence of a dimer-trimer equilibrium, in consideration of the observation that SDS PAGE of the enzyme gave a single 70 kDa band [84]. Since it is now well known that AMPD1 is routinely isolated from fresh rat muscle in a proteolysed form, as well as the enzyme from frozen rabbit muscle, we can reasonably advance the hypothesis that a high KCl concentration stabilizes the tetrameric structure of rat AMPD1 given by the association of two HPRG subunits to two proteolysed catalytic subunits.

The role of phosphate in stabilizing the AMPD1-HPRG complex is evidenced by the results of the sedimentation experiments with EDTA-treated rat AMPD1. As shown in Figure 3C, box A, the inactive apoenzyme sedimented as a single symmetrical peak of 254 kDa molecular weight in the presence of phosphate, and was fully reactivated by the addition of Zn^2+^. When the sedimentation of the apoenzyme was carried out at 0.5 M KCl, but in the absence of phosphate (Figure 3C, box B), the protein profile showed the presence of an asymmetrical peak of 193 kDa molecular weight, that coincides with the peak of the activity restored by Zn^2+^, but also gave evidence of the separation of an additional proteic component of lower molecular weight, void of activity, that could possibly be ascribed to the HPRG component [84]. It can also be inferred that the presence of high K^+^ concentration does not prevent the dissociation of HPRG from the apoenzyme. When the EDTA-inactivated enzyme sedimented in the absence of phosphate at low K^+^ concentration (0.02 M KCl) the separation between the different subunits of the AMPD1-HPRG complex became more marked as the position of the protein peak shifted to approx. 125 kDa molecular weight (Figure 3C, box C).

We have previously used Zn-affinity chromatography under denaturing conditions to separate the catalytic subunit of rabbit AMPD1, which was not retained by the resin and precipitated immediately after its elution in the flow-through from the HPRG component that was specifically retained on the column [43]. A quite different observation was made when rabbit AMPD1 was applied to the Zn-charged column equilibrated with 0.5 M NaCl/20 mM sodium phosphate (pH 7.0). Under these conditions, the whole protein was retained by the column. A slow elution of a fraction (40% of the total protein) with relatively high AMPD activity, that by SDS/PAGE showed the presence of a major 85 kDa component and a minor 70 kDa component similarly to the applied enzyme, was obtained by elution with 50 mM imidazole-HCl (pH 6.5) containing 0.5 M KCl [43]. Subsequent stripping elution with an EDTA-containing phosphate buffer eluted from the column a sharp protein peak containing the same 85 kDa and 70 kDa components but with low enzyme activity (30% of the total protein, but accounting for about 1% of the enzyme activity applied to the column), suggesting that in the presence of phosphate the protein-protein complex formed between the catalytic subunit and the HPRG component maintains its stability, even after the adsorption of HPRG to the Zn-charged column, in contrast to what is observed in the absence of phosphate. The EDTA-eluted fraction showed a higher stability during storage and a hyperbolic kinetics, that may be ascribed to the protection against loss of activity exerted by its higher HPRG content. Since the alteration of stability of rabbit AMPD1 observed when the molar ratio HPRG/AMPD was reduced is similar to that described for rabbit AMPD3 in the absence of phosphate [105], we can reasonably assume that the stabilizing effects of phosphate on cardiac AMPD3 could be explained by the protection exerted by the ion against loss of the HPRG component of the enzyme.

## 7. Intracellular Distribution Data of Striated Muscle AMPD. Evidence of the Interaction of the Enzyme with the Thin Filament

Available data indicate that AMPD is not an entirely soluble enzyme. For example, there is a measurable change in the distribution of AMPD1, from predominantly soluble (85–90% of activity) in resting rat skeletal muscle to the insoluble (up to 50–60% of activity) fraction during exercise [12,70,71]. Interaction with the cytoplasmic side of the erythrocyte membrane changes the solubility of the human AMPD3 isoform and reduces its catalytic activity. Moreover, the N-terminal region of the AMPD3 polypeptide is essential for this potent inhibition [26,27].

Based on the early reports that AMPD is a persistent contaminant of actomyosin preparations [102], several investigators have established that the AMPD activity is associated with various elements of contractile fibers. The reversible interaction between AMPD and myofibrils is promoted in vivo by intense muscle contraction, and results in an increase in AMPD activity, suggesting that the enzyme activation may help to maintain the adenylate charge and preserve cell viability under stressful conditions [11,12,70]. AMPD has been found to bind in vitro native myosin and purified myosin S2 fragments [107,108]. More recently, Mahnke-Zizelman and Sabina [69] reported that the binding capacity of AMPD isoforms to actomyosin resides in the C-terminal region of the enzyme, but that N-truncated forms of AMPD1 exhibits a significantly lower capacity to bind actomyosin, raising the question whether the AMPD interaction data previously described were affected by the degree of N-terminal proteolysis in the preparations. Somewhat different observations were obtained when the localization of AMPD in skeletal muscle was identified by immunostaining methods with both isolated myofibrils and muscle fibers grown in culture. In unstretched chicken and human muscle myofibrils, AMPD is bound to the lateral ends of the A-band extending into the I-band [41,109]. In addition, AMPD staining appears as a thin line in the center of the A-band, in correspondence to the M-line [41] where creatine kinase and adenylate kinase reside [110]. In agreement with those data, in rabbit fast-twitch muscles, the AMPD immunostaining was predominantly located in the A-band in a region significantly beyond the end of the myosin filament. It has also been suggested that AMPD1 may be attached to the myofibrils through titin molecules, and that this immobilization could improve its metabolic efficiency [111,112]. However, no clear influence on AMPD1 activity has been found as a result of the interaction of the enzyme with either titin or myosin.

We have recently demonstrated that HPRG is preferentially localized at the I-band level, where it shows the same distribution of actin and where AMPD1 is present in major concentration [52]. Based on our preliminary data that actin fragments could be isolated following the limited tryptic proteolysis of the isolated HPRG component of rabbit AMPD1 [113], we hypothesize that HPRG could be associated with the actin filament, and that the HPRG-AMPD1 complex could interact with proteins that take part in the constitution of the thin filament. The observation that HPRG binds with high affinity and in a Zn^2+^ or pH-dependent manner to tropomyosin present on the surface of the FGF-2-activated HUVEC cell [114] supports our hypothesis. Secondly, an interaction between HPRG and a component of the troponin complex, namely TnT, could also be hypothesized. The N-terminal region of fast skeletal TnT contains a putative zinc-binding site [7]. Interestingly, it has been reported that the AMPD1 allosteric properties, removed by limited proteolysis of the N-terminal region of the enzyme, can be restored by TnT or the phosphorylated N-terminal region of TnT [9], allowing one to suppose that an interaction between the N-terminal Zn-binding regions of AMPD1 and TnT could also occur in vivo [7]. In this light, it could also be hypothesized that the unrestrained AMPD1 activity that follows the N-terminus proteolytic cleavage of the enzyme in strenuously exercised muscle could be further regulated by the behavior of the HPRG component of AMPD1 as zinc metallochaperone, that might enhance the in vivo AMPD1 stability through insertion of zinc, essential for its activity, or modulating its intracellular availability [10,13,43].

Several intriguing correlations between AMPD and TnT are worth noting. Similarly to the three AMPD isoenzymes, three homologous TnT genes encode, in higher vertebrates, for a large number of TnT isoforms that are differentially expressed in fast skeletal, slow skeletal, and cardiac muscle fibers. These isoforms differ mainly in the sequence, the length, and the charge of the N-terminal region, while the central and C-terminal regions are highly conserved [115]. As seen for AMPD1 isoform, an in vivo calpain-induced degradation was observed with cardiac and skeletal muscle TnT. Restricted proteolysis of cardiac TnT by calpain occurs during physiological and pathological adaptation of cardiac muscle, such as ischemia– reperfusion and pressure overload [116]. Upon the selective removal of the 71 aa N-terminal variable region of cardiac TnT by m-calpain, the binding affinities of the protein for Tropomyosin, Troponin C, Troponin I were altered and a lower myosin ATPase activity, and myofibril force generation were observed [117].

Moreover, physio-pathological changes of striated muscle induced similar compensatory isoform switch for TnT and AMPD. The cardiac isoform of troponin T (cTnT) that is expressed in developing skeletal muscles is upregulated after sciatic nerve denervation and in various neuromuscular diseases [118]. In rodent skeletal muscle atrophy deriving from systemic diseases, which is characterized by a preferential loss of fast-twitch fibers, many genes required for ATP production and glycolysis are down-regulated, whereas some genes (including *Ampd3*) are dramatically up-regulated [119]. A change of subunit composition of AMPD was also observed in rat skeletal muscle in response to changes in *Ampd1* and *Ampd3* gene expression induced by denervation [61]. In skeletal muscle of the patients with Acquired AMPD deficiency, the consequent dilution of the AMPD1 immunohistochemical signal would be accompanied by a stabilizing effect on HPRG due to the association of the protein with either AMPD1 or AMPD3 component of the hybrid enzymes [62].

More recently, a non-canonical role for fast-twitching skeletal muscle TnT was reported by Zhang et al. [120]. TnT is fragmented in aging mice and both the full length TnT and the C-terminal domain of TnT can shuttle to the nucleus. Here, the expression of the voltage sensor Ca^2+^ channel a1 subunit, partially responsible for the loss of muscle strength during aging, is increased in the presence of TnT but is decreased by its C-terminal fragment.

Similarly to TnT, we have recently reported a nuclear localization of HPRG in the human skeletal muscle fiber [52]. Preliminary unpublished results of experiments carried out by transmission electron microscopy using the double immunogold staining for HPRG and AMPD1 give evidence a co-labeling of the two proteins at nuclear level, in the heterochromatin.

Further studies will be necessary to clarify whether the above reported similarities of TnT and AMPD in isoform switch during muscle development and adaptation, restricted proteolysis by calpain and nuclear localization are phenomena shared by the two proteins with other muscular proteins or indicate a distinctive relationship between the role and fate of TnT and AMPD.

## 8. Conclusions

It was previously reported that plasma HPRG associates with rabbit AMPD1 that can be assumed to represent the white-muscle AMPD isoform. The hypothesis that HPRG is involved in the formation of a heterotetramer with the AMPD1 catalytic subunits is supported by the experimental evidence that mammalian AMPD1 is stabilized by the presence of a molar concentration of KCl or a millimolar concentration of inorganic phosphate, conditions that maintained the association to HPRG, whereas the solubility of the enzyme is markedly reduced when the HPRG component is separated by zinc-affinity chromatography. Moreover, the observation that the HPRG-enriched AMPD1 undergoes a reduced rate of the fragmentation with storage suggests that the HPRG component might have a role in preserving the molecular integrity of the enzyme.

Although further studies are necessary, we speculate that HPRG might be a component of the cardiac and erythrocyte AMPD3 isoform as well, on the basis of the evidence that the absence of phosphate irreversibly converts cardiac AMPD3 into a molecular species with an atypical enzymatic behavior and an increased instability, similar properties being shown by human AMPD1 and AMPD3 isoforms produced with recombinant expression (i.e., in the absence of HPRG).

We have reported that the properties of rabbit AMPD1 are modified by the interaction with the N-terminus of TnT. The preferential localization of HPRG in the thin filament, where it shows the same distribution of actin and where AMPD1 is present in major concentration, strengthens the hypothesis that the HPRG-AMPD1 complex could play a physiological role in muscle contraction and metabolism through its interaction with proteins that take part in the constitution of the thin filament.

To date, the physiological role of HPRG in plasma has not clearly been determined, as it appears to be involved in many processes, such as blood coagulation and fibrinolysis, immune complex clearance, cell adhesion, cell migration, and transport of metal ions [47]. The observation that HPRG is acquired from plasma by many cells, including the skeletal muscle fibers [46,48], suggests that it might have also intracellular roles. Further studies will be necessary to establish whether the putative role of HPRG described in this review (as a metallochaperone promoting the association of AMPD1 to TnT) might be extended to other proteins via the Zn-binding domains present in them. Mice lacking the *Hprg* gene were viable and fertile but had no HRG in their blood. While (*Hprg*^−/−^) mice had significantly higher numbers of monocytes in their blood than wild-type mice, no other hematological parameters were altered. Measurements of antithrombin activity, prothrombin time and bleeding time suggested a mild anticoagulant and antifibrinolytic action of plasma HPRG. It would be of great interest to use this knockout model to further explore the role of the HPRG in tissues other than plasma, in particular whether the lack of HPRG would impair the activity of AMPD during muscle contraction [121].

## Figures and Tables

**Table 1:** Comparison of reported and predicted subunit molecular mass of striated muscle AMP deaminase (AMPD) isoforms in different species.

Species	RABBIT		RAT		CHICKEN	
Isoform	Reported (kDa)	Predicted (kDa)	Reported (kDa)	Predicted (kDa)	Reported (kDa)	Predicted (kDa)
AMPD1 (M)	80–85 ^a^ 68 ^b^–70 ^c^	86	60 ^d^ 68 ^e^–70 ^f^	86.3–85.9	69 ^b^–70 ^c^	85.1–85.6
AMPD3 (E)	80–81 ^g^	93.9	n.d.	88.4–92	69 ^h^–70 ^i^	88.8–93.6

^a^ [44]; ^b^ [87]; ^c^ [88]; ^d^ [90,91]; ^e^ [89]; ^f^ [84]; ^g^ [92,93]; ^h^ [94]; ^i^ [95].
